# Supplementary material for: The Construction and Exploration of a Comprehensive MicroRNA Centered Regulatory Network in Foxtail Millet (Setaria italica L.)
Source: Front Plant Sci. 2022 May 6;13:848474. doi: 10.3389/fpls.2022.848474 (PMC9121102; doi:10.3389/fpls.2022.848474)
Supplement: Supplementary file 5 [file Data_Sheet_5.zip › Sit-miR396c_Seita.9G156600.1_885_TPlot.pdf]

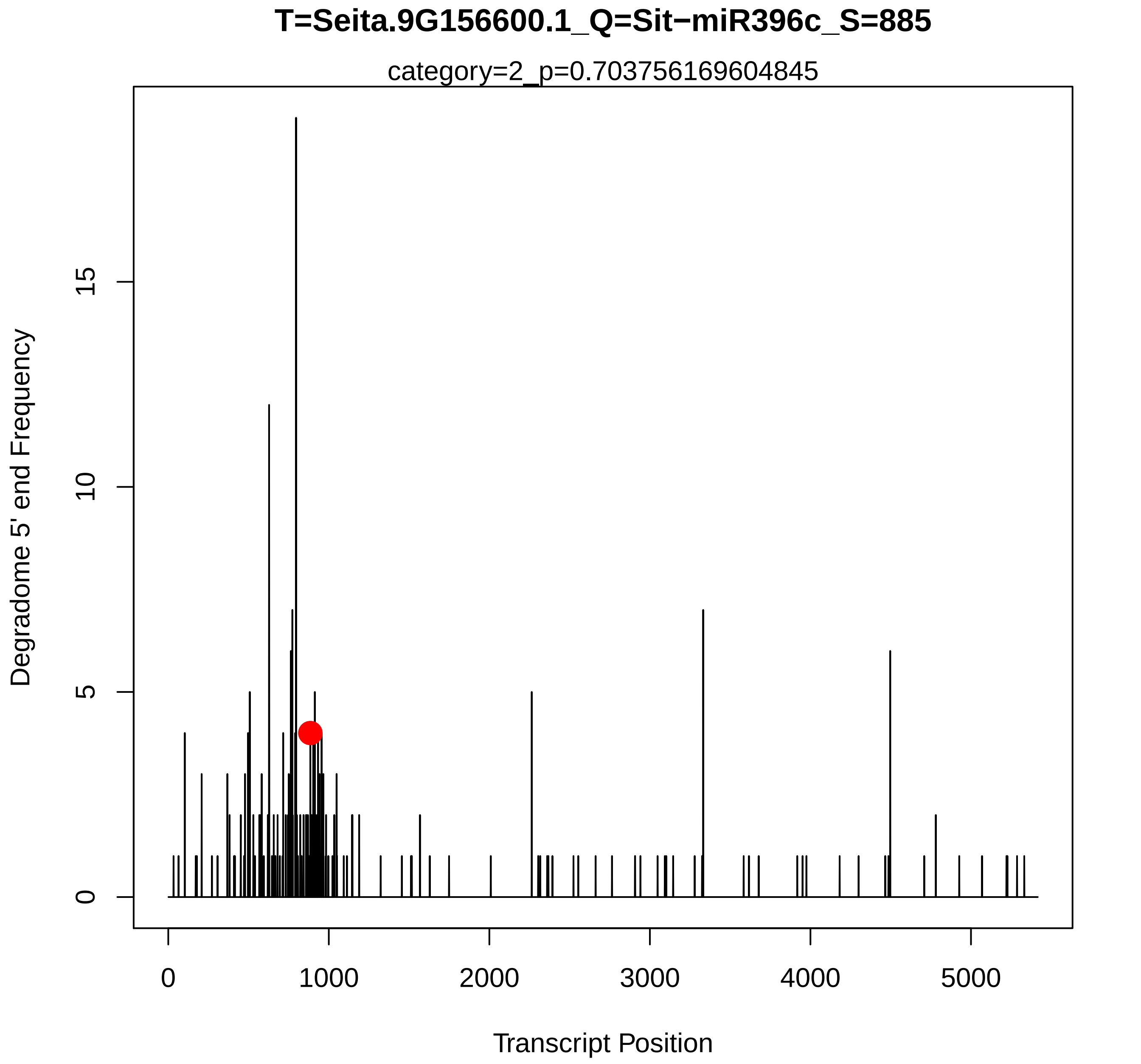
T=Seita.9G156600.1_Q=Sit−miR396c_S=885
category=2_p=0.703756169604845
Degradome 5' end Frequency
0
5
10
15
Transcript Position
0
1000
2000
3000
4000
5000
